# Variable expressivity of *FGF3 *mutations associated with deafness and LAMM syndrome

**DOI:** 10.1186/1471-2350-12-21

**Published:** 2011-02-09

**Authors:** Saima Riazuddin, Zubair M Ahmed, Rashmi S Hegde, Shaheen N Khan, Idrees Nasir, Uzma Shaukat, Sheikh Riazuddin, John A Butman, Andrew J Griffith, Thomas B Friedman, Byung Yoon Choi

**Affiliations:** 1 Laboratory of Molecular Genetics, Division of Pediatric Otolaryngology Head & Neck Surgery, Cincinnati Children's Hospital Research Foundation, and the University of Cincinnati, College of Medicine, Cincinnati, OH, USA; 2 Division of Pediatric Ophthalmology, Cincinnati Children's Hospital Research Foundation, and the University of Cincinnati, College of Medicine, Cincinnati, OH, USA; 3 Division of Developmental Biology, Cincinnati Children's Hospital Medical Center and the University of Cincinnati College of Medicine, Cincinnati, OH, USA; 4 National Center of Excellence in Molecular Biology, University of the Punjab, Lahore, Pakistan; 5 Allama Iqbal Medical College-Jinnah Hospital Complex, University of Health Sciences, Lahore, Pakistan; 6 Diagnostic Radiology Department, The Clinical Center, National Institutes of Health, Bethesda, MD, USA; 7 Otolaryngology Branch, National Institute on Deafness and Other Communication Disorders, National Institutes of Health, Rockville, MD, USA; 8 Laboratory of Molecular Genetics, National Institute on Deafness and Other Communication Disorders, National Institutes of Health, Rockville, MD, USA; 9 Department of Otorhinolaryngology, Seoul National University College of Medicine, Seoul National University Bundang Hospital, Seongnam, Republic of Korea

## Abstract

**Background:**

Recessive mutations of fibroblast growth factor 3 (FGF3) can cause LAMM syndrome (OMIM 610706), characterized by fully penetrant complete labyrinthine aplasia, microtia and microdontia.

**Methods:**

We performed a prospective molecular genetic and clinical study of families segregating hearing loss linked to *FGF3 *mutations. Ten affected individuals from three large Pakistani families segregating *FGF3 *mutations were imaged with CT, MRI, or both to detect inner ear abnormalities. We also modeled the three dimensional structure of FGF3 to better understand the structural consequences of the three missense mutations.

**Results:**

Two families segregated reported mutations (p.R104X and p.R95W) and one family segregated a novel mutation (p.R132GfsX26) of *FGF3*. All individuals homozygous for p.R104X or p.R132GfsX26 had fully penetrant features of LAMM syndrome. However, recessive p.R95W mutations were associated with nearly normal looking auricles and variable inner ear structural phenotypes, similar to that reported for a Somali family also segregating p.R95W. This suggests that the mild phenotype is not entirely due to genetic background. Molecular modeling result suggests a less drastic effect of p.R95W on FGF3 function compared with known missense mutations detected in fully penetrant LAMM syndrome. Since we detected significant intrafamilial variability of the inner ear structural phenotype in the family segregating p.R95W, we also sequenced *FGF10 *as a likely candidate for a modifier. However, we did not find any sequence variation, pointing out that a larger sample size will be needed to map and identify a modifier. We also observed a mild to moderate bilateral conductive hearing loss in three carriers of p.R95W, suggesting either a semi-dominant effect of this mutant allele of *FGF3*, otitis media, or a consequence of genetic background in these three family members.

**Conclusions:**

We noted a less prominent dental and external ear phenotype in association with the homozygous p.R95W. Therefore, we conclude that the manifestations of recessive *FGF3 *mutations range from fully penetrant LAMM syndrome to deafness with residual inner ear structures and, by extension, with minimal syndromic features, an observation with implications for cochlear implantation candidacy.

## Background

Sensorineural hearing loss is one of the most common congenital disorders, affecting at least 1 in 1,000 births [1,2]. Up to 39% of sensorineural deafness is associated with radiologically detectable inner ear malformations [3-5]. Among those anomalies, complete labyrinthine aplasia (CLA), also known as "Michel aplasia," is reported to comprise 1% of cochlear bony abnormalites [6]. CLA merits special consideration since it precludes cochlear implantation due to the lack of a spiral ganglion and cochleovestibular nerve fibers [7-10]. CLA has been reported in association with other anomalies [11,12] including thalidomide embryopathy [11], microtia and microdontia [13]. Until recently, the etiology of CLA was unknown except for cases associated with thalidomide exposure [11].

Mendelian inheritance was initially suggested for some cases of CLA, but no genetic linkage data were reported [14,15]. Tekin and co-authors subsequently described an autosomal recessive deafness phenotype, now referred to as LAMM syndrome (OMIM 610706), comprising CLA, microtia and microdontia [9]. LAMM syndrome co-segregated with recessive mutations of the gene encoding fibroblast growth factor 3 (FGF3; OMIM 164950) on chromosome 11q13.2-q13.3 [9,10]. A total of five Turkish families and one Saudi family segregating LAMM syndrome and recessive *FGF3 *mutations have been reported to date [7,9,10]. All of the affected individuals had fully penetrant LAMM syndrome with CLA, microdontia, and type I microtia in which the auricle has a dysmorphic helix and antihelix but different parts of the auricle are still recognizable. In addition, temporal bone CT images of 14 affected members of the previously reported families revealed complete labyrinthine aplasia, although a cystic vestibulum was detected once [7,9,10]. Ramsebner et al. (2009) recently reported a missense *FGF3 *mutation (p.R95W) associated with less severe phenotypes, clinically distinct from those of LAMM syndrome [16]. However, it was not clear whether this mild phenotype in the Somali family is due to the mutation, genetic background or both.

Mice deficient for FGF3 do not model LAMM syndrome but rather have abnormal inner ears with variable penetrance and expressivity on a uniform genetic background [17-19]. In mice, *Fgf3 *is expressed in the otic vesicle and in the adjacent hindbrain during otic placode induction and subsequent early inner ear morphogenesis [18,20]. Loss-of-function data from mice suggest that FGF3 affects molecular patterning of the dorsal otocyst and dorsal otic gene expression that is induced by WNT signals, but it does not critically influence ventrally expressed otic genes important for cochlear development [18].

Here we report three Pakistani families co-segregating profound recessive deafness with mutant alleles of *FGF3*. In one family, a recessive p.R95W mutation was associated with a variable inner ear and auricular phenotype,, similar to the reported mild phenotype of this allele [16]. Unlike in the Somali family, significantly milder dental phenotypes were also observed in this Pakistani family. Therefore, we conclude that the phenotypic spectrum of recessive *FGF3 *mutations range from fully penetrant LAMM syndrome to deafness with residual inner ear structures and absent or minimal syndromic features. We also suggest that p.R95W might exert a semi-dominant effect upon the function of *FGF3*.

## Methods

### Subject enrollment

This study was approved by the Institutional Review Board (IRB) at the National Centre of Excellence in Molecular Biology (NCEMB), Lahore, Pakistan (FWA00001758) and the Combined Neuroscience IRB at the National Institutes of Health, USA (OH-93-N-016). Written informed consent was obtained from adult subjects and parents of minor subjects. We ascertained five Pakistani families (PKDF537, PKDF702, PKDF295, PKDF817 and PKDF887) segregating profound hearing loss significantly linked to a 4.81-cM interval on human chromosome (11q13.2-q13.3), which was designated as the *DFNB63 *locus [21]. Four families were previously reported [21] and one family (PKDF887) was identified through a subsequent screen with microsatellite markers (*D11S4113*, *D11S4136 *and *D11S4162*) linked to this locus. We obtained clinical data only on the auditory phenotype when these families were initially ascertained. Genomic DNA from affected members of the five families was extracted from 10 ml of peripheral venous blood as described [22]. *FGF3 *is linked to *LRTOMT*, in which recessive mutations are associated with non-syndromic deafness DFNB63 [23,24]. Among our five study families segregating deafness linked to chromosome 11q13.2-q13.3, one family (PKDF702) was shown to have recessive mutations in *LRTOMT *[23].

### Mutation screening of FGF3 and 10

Primers for polymerase chain reaction (PCR) amplification and *FGF3 *and *FGF10 *sequencing were designed using Primer3 (http://frodo.wi.mit.edu/primer3/). Methods for direct sequencing of PCR products were described previously [22]. BigDye terminator reaction products were resolved on an ABI3730 instrument. Sequencing traces were analyzed with the SeqMan Pro tool of DNASTAR Lasergene software (http://www.dnastar.com). *FGF10 *was screened since it was considered to be a candidate modifier of *FGF3*.

### Molecular modeling

To better understand the structural consequences of p.R95W that we detected in this study, we modeled the three-dimensional structure of FGF3 using the SWISS-MODEL server [25] and FGF10, an orthologue with the greatest sequence identity and with available crystallographic coordinates as a template. This model was then docked on FGFR2b in a manner similar to that seen in the FGF10-FGFR2b complex (1NUN.PDB) [26].

### Phenotype analysis

After the detection of mutations of *FGF3*, affected and unaffected family members were clinically re-evaluated. Hearing was evaluated by pure tone audiometry at octave frequencies with intensities up to 100 dB HL. Facial nerve and vestibular function was evaluated by facial expressions and tandem gait/Romberg testing, respectively. We applied the staging system of microtia described by Weerda et al. [27]. Computed tomography (CT) and magnetic resonance imaging (MRI) of the temporal bones were performed, when possible, to examine middle and inner ear structures, and the internal auditory canal and its contents in affected individuals. The MR images were acquired with a 1.5-T system and a T2-weighted fast spin-echo sequence (repetition time, 3920 ms; echo time, 94 ms; slice thickness, 3 mm) and/or 3D fast imaging with steady state acquisition (3D FIESTA) (repetition time, 7 ms; echo time, 3 ms, slice thickness, 0.8 mm).

## Results

### *FGF3 *mutations in DFNB63-linked families

**Figure 1:**
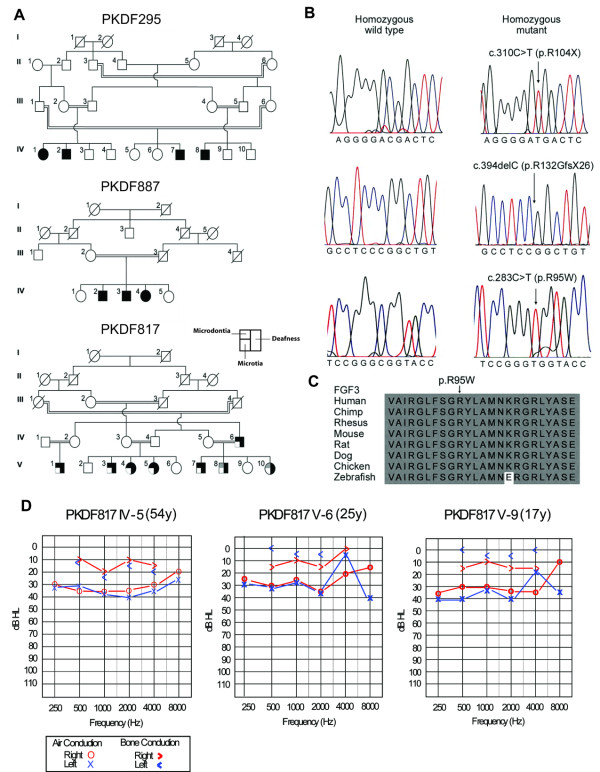
**Pedigrees, mutations and clinical data**. (A) Pedigrees of Pakistani families segregating prelingual hearing loss and mutations of *FGF3*. The maximum LOD scores for these families are 4.26 [21], 2.5 [21] and 4.87, respectively. Individuals with less severe phenotypes are shown with grey shading. (B) Wild type and mutant alleles of *FGF3 *from unaffected and affected members of families PKDF295, PKDF887 and PKDF817. (C) ClustalW alignment of FGF3 amino acid residues 86-109 shows that Arginine residue at position 95 (p.R95, arrow) is conserved in a variety of species. The mutated amino acid residue in human FGF3 was numbered according to NM_005247.2 (cDNA) and NP_005238 (protein). (D) Pure tone audiometry results for three heterozygous carriers of p.R95W who show a mild to moderate degree of conductive hearing loss with air-bone conduction threshold gaps that range from 20 to 35 dB.

We identified homozygous *FGF3 *mutations co-segregating with deafness in three families (PKDF295, PKDF817 and PKDF887). Families PKDF537 and PKDF702 did not segregate *FGF3 *mutations. One mutation (p.R132GfsX26 (c.394delC) from family PKDF887) was novel and the other two mutations (p.R95W (c.283 C > T) from PKDF817 and p.R104X (c.310C > T) from PKDF295) were previously reported (Table 1; Figure 1) [9,16]. These mutations were not found in 162 ethnically matched Pakistani normal-hearing control individuals, indicating that the variants are not common polymorphisms. The p.R132GfsX26 mutation is predicted to produce a frameshift at codon 132 followed by 25 missense amino acids and premature termination, resulting in the truncation of one third of the FGF3 protein. The p.R95W associated with the milder phenotype affects a residue that is highly conserved in known FGF3 orthologs (Figure 1). Since we detected significant intrafamilial variability of the inner ear structural phenotype in the family segregating p.R95W (see below), we also sequenced *FGF10 *as a likely candidate for a modifier but could not find any mutation in *FGF10 *from family PKDF817.

**Table 1:** Summary of clinical findings of individuals carrying homozygous *FGF3 *mutations

*Family No.*	*Ethnicity*	*Subject No.*	*Sex*	*Type I microtia*	*Microdontia with conical teeth*	*CT or MRI findings of inner ear*	*Nucleotide change^b^*	*Effect^b^*	*Reference*
PKDF 817	Pakistani	V:1	M	Normal	+	Bi-CLA	c.283C > T	p.R95W	[16]
		V:3	M	Normal	+	**Rt**-dysplastic SCC, bony IAC(+), single cochleovestibular nerve **Lt**-One and a half turn of cochlea, short broad cystic LSC confluent with the vestibule, **bony IAC(+)**			
		V:4	F	Normal	+	Bi-CLA			
		V:5	F	Normal	+	Not available			
		V:7	M	Normal	+	**Rt**-Reminiscent of cochlear basal turn, **Lt**-CLA			
		V:8	M	Normal	Equivocal	Bi-CLA			
		V:10	F	Normal	Equivocal	Bi-CLA			
PKDF 295	Pakistani	IV:1	F	+	+	Bi-CLA Bi- Subarachnoid cyst	c.310C > T	p.R104X	[9]
		IV:2	M	+	+	Bi-CLA Bi- Subarachnoid cyst			
		IV:7	M	+^a^	+^a^	Not available			
		IV:8	M	+^a^	+^a^	Not available			
PKDF 887	Pakistani	IV:2	M	+	+	Bi-CLA Bi-Subarachnoidal cyst	c.394delC	p.R132GfsX26	this study
		IV:3	M	+	+	Bi-CLA Bi- Subarachnoid cyst			
		IV:4	F	+^a^	+^a^	Not available			
*Somali family*	*Somali*	3	F	+	+	Rt- One and a half turn of cochlea, Lt-CLA	c.283C > T*	p.R95W	[16]
		5	M	+	+	Bi-CLA			
		7	F	Normal	+	Rt-CLA Lt-Common cavity			
		8	F	+	+	Bi-CLA			
1	Turkish	1	F	+	+	Bi-CLA	c.255delT	p.I85MfsX15	[10]
2	Turkish	IV:1	F	+	+	**Lt**-CLA, **Rt**-Rudimentary cystic vestibule, but no bony IAC Bi-Subarachnoid cyst	c.17T > C	p.L6P	[10]
		IV:2	M	+	+	Bi-CLA			
		IV:3	M	+	+	Bi-CLA			
3	Turkish	1	M	+	+	Bi-CLA	c.466T > C	p.S156P	[9]
		2	M	+	+	Bi-CLA			
		3	M	+	+	Bi-CLA			
		4	M	+	+	Bi-CLA			
		5	F	+	+	Bi-CLA			
4	Turkish	1	M	+	+	Bi-CLA	c.310C > T	p.R104X	[9]
		2	M	+	+	Bi-CLA			
		3	M	+	+	Bi-CLA			
5	Turkish	1	F	+	+	Bi-CLA	c.616delG	p.V206SfsX13	[9]
1	Saudi	VI:8	F	+	+	**Rt**-CLA, **Lt**-rudimentary cystic vestibule, but no bony IAC	c.196G > T	p.G66C	[7]
		VI:9	M	+	+	Not available			
		IV:3	M	+	+	Bi-CLA Bi- Subarachnoid cyst			
		IV:4	F	+^a^	+^a^	Not available			

^a^Inferred from observations by family members.

^b^Mutations numbered according to NM005247.2 (cDNA) and NP_005238 (protein).

*Ramsebner et al. (2009) reported this allele as c.284C > T, but the wild type nucleotide at 284 is a G.

The affected homozygotes are profoundly deaf (data not shown). Interestingly, in contrast to heterozygous carriers from PKDF295 and PKDF887, all of the three tested heterozygous carriers of p.R95W in family PKDF817 (PKDF817 IV-5, V-6 and V-9) showed a mild to moderate degree of bilateral conductive hearing loss (Figure 1D), while having a normal auricular and dental phenotype (data not shown). However, we cannot rule out otitis media in these carriers due to a lack of tympanometric data. The variant (c.283C > T) associated with the variable phenotype has no predicted effect upon splicing using ESEfinder v3.0 (http://rulai.cshl.edu/cgi-bin/tools/ESE3/esefinder.cgi?process = home) or BDGP (http://www.fruitfly.org/seq_tools/splice.html) programs. Therefore, it is unlikely that this variant creates a cryptic splice site or perturbs an exonic splice enhancer site that would lead to a leaky splice mutation.

### Auricular and dental phenotype

The clinical findings for our subjects, Somali subjects homozygous for p.R95W, and the previously reported LAMM subjects [7,9,10,16] are summarized in Table 1. Whereas all of the affected individuals with homozygous *FGF3 *mutations from our three study families are profoundly deaf, they had variable auricular and dental anomalies (Table 1). We did not observe facial nerve dysfunction in any of the affected subjects.

**Figure 2:**
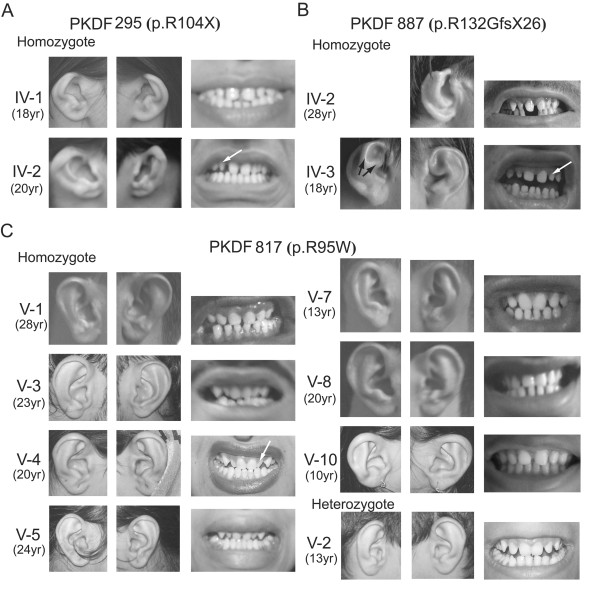
**Auricular and dental findings of affected members of families with recessive *FGF3 *mutations**. (A) Type I microtia with shortening of the superior portions of the auricles, overfolded helices and anteverted pinnae are shown as well as microdontia with conical, sharp and pointed lateral incisors (white arrows) in PKDF295 IV-1 and IV-2. (B) Type I microtia with shortening of the superior portions of the auricle caused by incomplete formation of the upper helix and helical crus. The antihelix and antihelical crura (black arrow) are relatively well developed. (C) Normal auricles of deaf individuals from family PKDF817. Some individuals, especially V-8 and V-10, show a milder dental phenotype compared with those in families PKDF295 and PKDF887.

There was incomplete formation of the superior helix and helical crus of affected members of families PKDF295 and PKDF887. This resulted in shortened auricles with indiscernible cymba conchae, compatible with type I microtia (Figure 2A-B). We detected no distinct abnormal auricular findings in most of the affected p.R95W homozygotes from family PKDF817 (Figure 2C). Affected members of families PKDF295 and PKDF887 showed microdontia with conical, sharp and pointed lateral incisors (white arrow, Figure 2) and widely spaced teeth. In contrast, many of the affected members of PKDF817 had a subtle dental phenotype (Figure 2C). PKDF817 V-3, V-4 and V-5 individuals did not show wider interdental distances compared with those from a heterozygous carrier of the missense mutation. Individuals V-8 and V-10 from family PKDF817 showed neither malformed lateral incisors nor unusually wide inderdental spaces (Figure 2C).

### Inner ear phenotype

**Figure 3:**
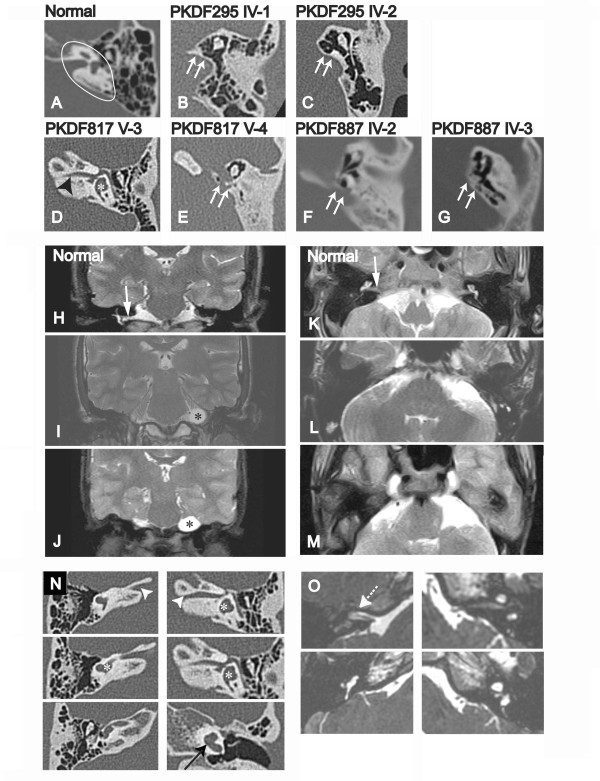
**Radiological images from affected individuals with *FGF3 *homozygous mutations**. Images (A through G) are axial temporal bone CT sections. (A) Inner ear structures (Internal auditory canal (IAC), cochlea, and vestibule) in petrous bone are clearly seen (white ellipsoid) in a normal subject. (B, C, E, F, and G) Complete labyrinthine aplasia with a flat medial wall (white arrows) of the middle ear cavity. (D) A narrow IAC (black arrowhead), posterior semicircular canal and vestibule (white asterisk) are visible. Images (H-J and K-M) are coronal and axial sections, respectively, of T2-weighted MR images (T2WI) of the cerebellopontine angle region. Affected individuals, PKDF295 IV-2 (I and L) and PKDF887 IV-3 (J and M) have a presumed subarachnoid cyst (black asterisk). The cochleovestibular nerve and inner ear structures (white arrow) detected in a normal subject (H and K) are not clearly seen in affected subjects (I, J and M). Images (N and O) are contiguous axial temporal bone CT sections and axial 3D-FIESTA MR images from PKDF817 V-3, respectively. (N) The osseous IAC (white arrowhead), vestibule (white asterisk), and 1-1/2 turns of the cochlea (black arrow) can be identified in the left ear. The vestibule and posterior semicircular canal are shown in the right ear. (O) The presence of a single cochleovestibular nerve (white dotted arrow) can be identified in the right side. Cochlea, vestibule and posterior semicircular canal are shown in the left ear.

We obtained temporal bone CT scans, T2-weighted/3D FIESTA MR images, or both from ten affected individuals (two from family PKDF295, two from PKDF887 and six from PKDF817) and three heterozygous individuals (one from each family). Heterozygous carriers of p.R104X (III-3, PKDF295), p.R132GfsX26 (III-1, PKDF887) and p.R95W (V-2, PKDF817) did not show any abnormal findings. The images demonstrated bilateral CLA in all four deaf members of PKDF295 and PKDF887 and in four of the six deaf members of PKDF817 (Table 1 and Figure 3). These subjects lacked all inner ear structures, including cochlear, vestibule, and all semicircular canals (white arrows, Figure 3B-C and 3E-G). The internal auditory canal (IAC) was also aplastic and the cochleovestibular nerve was not detected by T2-weighted MRI (Figure 3 panels I, L are from PKDF295 IV-2 while panels J, M are from PKDF887 IV-3). All affected individuals show normal middle ear development. However, the degree of mastoid and middle ear pneumatization was variable among affected individuals.

Partial development of the labyrinth was observed in two p.R95W homozygotes, including one (PKDF817 V-3) with a cochlear basal turn, vestibule, and posterior semicircular canal (Figure 3D and 3N from PKDF817 subject V-3), while the other four p.R95W homozygotes had no detectable inner ear structures (Table 1). Axial CT images showed the presence of bilateral IACs, albeit narrower than normal, especially on the left side (Figure 3N). The facial nerve and a single cochleovestibular nerve bundle were observed on the right side (arrow, Figure 3O), but only the facial nerve was detected in the left temporal bone in axial 3D-FIESTA MR images. Another affected member of this family (V-7) also had a rudimentary cochlear basal turn on the right side (Table 1). A probable subarachnoid cyst was detected in all four affected members of PKDF295 and PKDF887 with MR images available for review (Figure 3I-J), as previously described for another LAMM subject [10].

### Molecular modeling

**Figure 4:**
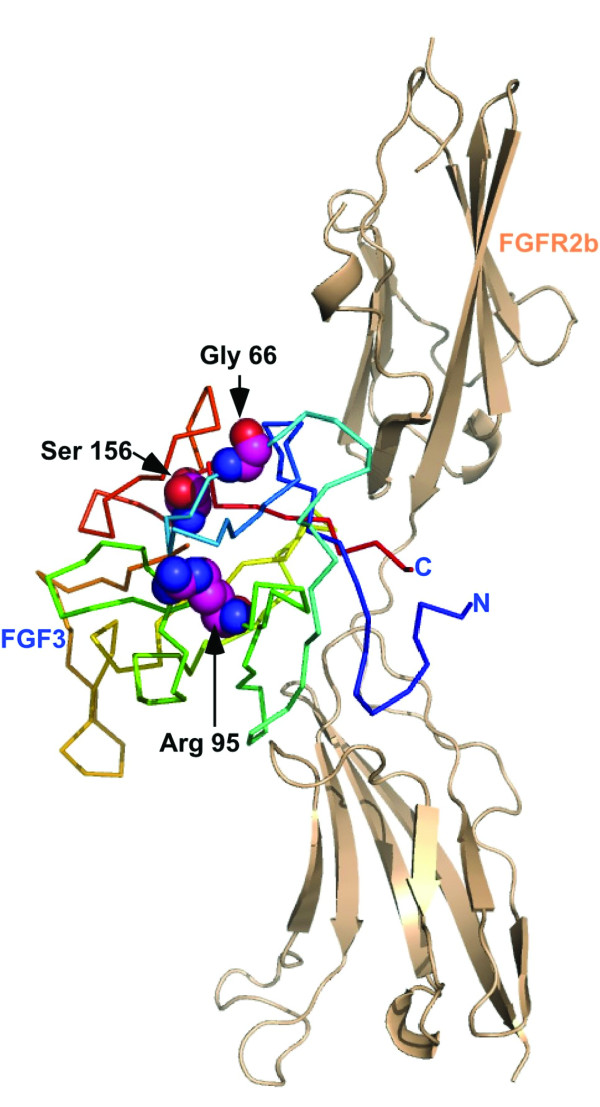
**A homology model of FGF3 generated using the structure of FGF10 (1NUN.PDB) as a template and the SWISS-MODEL server**. FGF3 was then docked on the structure of FGFR2b in a manner identical to that of FGF10 in the FGF10-FGFR2b complex. FGFR2b is represented by the tan cartoon, while FGF3 is shown as a ribbon color coded from the N- to the C-terminus in blue to red. The residues Gly 66, Arg 95 and Ser 156 are shown as spheres. The image was generated using PYMOL [25].

The common core of all FGFs consists of 140 amino acids that fold into a ß-trefoil domain. One surface of this domain is typically involved in heparan sulfate binding, while another participates in receptor (FGFR) binding. We mapped p.R95W, associated with a milder phenotype, onto the model of FGF3. Two other missense mutations (p.G66C [7] and p.S156P [9]) that were associated with fully penetrant LAMM syndrome were also mapped for comparison (Figure 4). Arginine (R) at residue 95 (Arg 95) is located away from both the FGFR2b receptor and heparan sulfate binding sites of the FGFs (Figure 4). While an arginine to tryptophan mutation is non-conservative, both side-chains have a hydrophobic section and the capacity to form hydrogen bonds. Given the partially buried nature of the R95 side chain, replacement of this residue with the more bulky tryptophan (W) could be accommodated with some degree of protein destabilization, but not a complete loss of activity. In contrast, the p.G66C mutation detected in subjects with fully penetrant LAMM syndrome introduces a partially buried cysteine (Cys) residue in FGF3. As a half cysteine, the reactivity of this thiol group is likely to lead to a reduced functional half-life for FGF3 (p.G66C), as has been well documented in analyses of other FGFs [28,29]. The other possibility is the formation of a disulfide bond, but the nearest cysteine at position 50 is too far to allow disulfide linkage without conformational changes. Interestingly a cysteine residue in FGF15/19 at the position structurally similar to Cys 66 in FGF3 forms a disulfide bond with a Cys 12 to its N-terminus [30]. While a similarly positioned cysteine is not present in FGF3, it is conceivable that an altered conformation could allow Cys 50 to be disulfide linked with Cys 66 of FGF3. With regard to p.S156P that was also detected in LAMM syndrome patients, serine at residue 156 (Ser 156) is located near the putative basic heparin binding site of the FGFs referred to as the glycine-box [31]. Structural studies have shown that FGF interaction with heparin can accommodate a large degree of sequence variation in the FGFs since much of the interaction involves van der walls contact with the protein backbone between the equivalent of residues 158 and 174 of FGF3 [32]. It is possible that p.S156P alters the peptide backbone in this region enough to affect binding of heparan sulfate and thus alter the stability of the FGF signalling complex.

## Discussion

Recessive *FGF3 *mutations associate with a broad spectrum of inner ear and craniofacial phenotypes that show inter- and intrafamilial variability. Our study confirms that at least some recessive mutations of *FGF3 *may not always result in fully penetrant LAMM syndrome. Furthermore, we noted a less prominent dental phenotype in subjects with p.R95W, in contrast to the severe dental phenotype reported from the Somali family [16]. Taken together, our results show that the manifestations of recessive *FGF3 *mutations can involve nearly non-syndromic deafness with variable inner ear structural development. In this regard, mutations of either *LRTOMT *[23] or *FGF3 *should be considered when incompletely characterized nonsyndromic deafness is found to be linked to genetic markers of the *DFNB63 *locus on chromosome 11q 13.2 -q13.3.

The less severe phenotype associated with p.R95W segregating in Pakistani family PKDF817 is probably not due solely to genetic background, since the milder phenotype of homozygous p.R95W individuals has also been reported in an unrelated family from Somalia. The amino acid substitution of p.R95W may have a milder pathogenic effect as compared with other mutations of *FGF*. Our molecular modeling results support the possibility of less severe pathogenic effects of this allele since this variant is predicted to affect neither FGFR2b receptor nor heparan sulfate binding sites. Alone or in combination with a less severe pathogenic potential of p.R95W, there might be a differential effect of mutant alleles of *FGF3 *on different target organs. While these molecular models provide a plausible rationale for the effects of the mutations identified here, there remains the caveat that available structural information does not account for the role that receptor glycosylation might play in FGF interaction. In addition, phenotypic variability within and between the affected individuals in these two families also indicates a role for environment, stochastic events, genetic background, or a combination of these influences. In affected members of family PKDF817, we could not find a variant in *FGF10*, a potential candidate for a modifier of *FGF3*. To address the presence of modifiers, if any, larger families segregating a milder phenotype and p.R95W would be required for linkage analyses.

Our identification of a mild to moderate conductive hearing loss in heterozygous carriers raises the question of whether p.R95W exerts a semi-dominant pathogenic effect upon the function of FGF3. These data warrant careful interpretation, since we were unable to obtain information about the status of the tympanic membrane and ossicles in carriers. We initially reasoned that this may reflect the same pathogenic mechanism underlying autosomal dominant lacrimo-auriculo-dento-digital syndrome (LADD syndrome; OMIM 149730) caused by heterozygous mutations of *FGFR2b *[33]. FGF3 is known to bind to FGFR2b with high affinity [34,35]. Some patients with LADD syndrome have been reported to show isolated mild conductive hearing loss [36], although many patients manifest a mild to moderate mixed type hearing loss. Moreover, the dental and auricular phenotypes from LADD syndrome patients are very similar with those in LAMM syndrome, while another autosomal dominant oto-dental syndrome (MIM 166750) recently reported in association with heterozygous microdeletions affecting *FGF3 *[37] shows significantly different dental phenotypes. In this regard, this possible semi-dominant phenotype might be consistent with the hypothesis that p.R95W affects the interaction between FGF3 and FGFR2 [16]. However, our molecular modeling results argue against this hypothesis, since p.R95 is located away from the FGFR2b binding surfaces of FGF3. Alternatively, an allele of a totally different gene accounts for the hearing loss in p.R95W heterozygous carriers in the inbred family PKDF817, as the Somali p.R95W heterozygotes were reported not to have hearing loss [16] The resolution of this question will require careful molecular and clinical examination of additional carriers of this allele and other mutations of *FGF3*. The inner ear phenotypic variability within families is consistent with the highly variable inner ear phenotype of *Fgf3 *knockout mice on a uniform genetic background [18,19] and functional redundancy of *Fgf3 *and *Fgf10 *in otic vesicle formation [17,38].

The options for auditory rehabilitation of LAMM patients with CLA are limited to vibrotactile hearing devices or brainstem implants. The presence of an osseous IAC and a cochleovestibular nerve has never been described in association with homozygous *FGF3 *mutations before this study. The presence of a cochleovestibular nerve and a cochlear remnant is a significant consideration for cochlear implantation candidacy. Because of intrafamilial variability, some patients with *FGF3*-related hearing loss are potential candidates for cochlear implantation, even if CLA has already been documented in other affected family members.

## Conclusions

We conclude that the manifestations of recessive *FGF3 *mutations range from fully penetrant LAMM syndrome to deafness with residual inner ear structures and, by extension, with minimal syndromic features.

## Competing interests

The authors declare that they have no competing interests.

## Authors' contributions

SR, ZMA, SNK, IN, US and BYC carried out the molecular genetic studies and participated in the sequence alignment. RSH carried out the molecular modeling. JAB, AJG and BYC carried out the interpretation of clinical data. SR, ZMA and BYC participated in the design of the study. SR, ZMA and BYC conceived of the study and drafted the manuscript. SR, AJG and TBF supervised this study and critically revised the manuscript for important intellectual content. All authors read and approved the final manuscript.

## Pre-publication history

The pre-publication history for this paper can be accessed here:

http://www.biomedcentral.com/1471-2350/12/21/prepub

## References

[B1] Morton CC Nance WE Newborn hearing screening--a silent revolution N Engl J Med 2006 354 20 2151 2164 10.1056/NEJMra050700 16707752

[B2] Smith RJ Bale JF Jr White KR Sensorineural hearing loss in children Lancet 2005 365 9462 879 890 10.1016/S0140-6736(05)71047-3 15752533

[B3] Bamiou DE Phelps P Sirimanna T Temporal bone computed tomography findings in bilateral sensorineural hearing loss Arch Dis Child 2000 82 3 257 260 10.1136/adc.82.3.257 10685935 PMC1718255

[B4] Mafong DD Shin EJ Lalwani AK Use of laboratory evaluation and radiologic imaging in the diagnostic evaluation of children with sensorineural hearing loss Laryngoscope 2002 112 1 1 7 10.1097/00005537-200201000-00001 11802030

[B5] Wu CC Chen YS Chen PJ Hsu CJ Common clinical features of children with enlarged vestibular aqueduct and Mondini dysplasia Laryngoscope 2005 115 1 132 137 10.1097/01.mlg.0000150691.85387.3f 15630381

[B6] Jackler RK Luxford WM House WF Congenital malformations of the inner ear: a classification based on embryogenesis Laryngoscope 1987 97 3 Pt 2 Suppl 40 2 14 3821363 10.1002/lary.5540971301

[B7] Alsmadi O Meyer BF Alkuraya F Wakil S Alkayal F Al-Saud H Ramzan K Al-Sayed M Syndromic congenital sensorineural deafness, microtia and microdontia resulting from a novel homoallelic mutation in fibroblast growth factor 3 (FGF3) Eur J Hum Genet 2009 17 1 14 21 10.1038/ejhg.2008.141 18701883 PMC2985964

[B8] Lindsay JR Profound childhood deafness. Inner ear pathology Ann Oto Rhinol Laryngology 1973 82 Suppl 5 1 121 4573293

[B9] Tekin M Hismi BO Fitoz S Ozdag H Cengiz FB Sirmaci A Aslan I Inceoglu B Yuksel-Konuk EB Yilmaz ST Homozygous mutations in fibroblast growth factor 3 are associated with a new form of syndromic deafness characterized by inner ear agenesis, microtia, and microdontia Am J Hum Genet 2007 80 2 338 344 10.1086/510920 17236138 PMC1785350

[B10] Tekin M Ozturkmen Akay H Fitoz S Birnbaum S Cengiz FB Sennaroglu L Incesulu A Yuksel Konuk EB Hasanefendioglu Bayrak A Senturk S Homozygous FGF3 mutations result in congenital deafness with inner ear agenesis, microtia, and microdontia Clin Genet 2008 73 6 554 565 10.1111/j.1399-0004.2008.01004.x 18435799

[B11] Jorgensen MB Kristensen HK Buch NH Thalidomide-Induced Aplasia of the Inner Ear J Laryngo Oto 1964 78 1095 1101 10.1017/s0022215100063234 14235573

[B12] Kavanagh KT Magill HL Michel dysplasia. Common cavity inner ear deformity Pediatr Radiol 1989 19 5 343 345 10.1007/BF02467313 2755750

[B13] Hersh JH Ganzel TM Fellows RA Michel's anomaly, type I microtia and microdontia Ear Nose Throat J 1991 70 3 155 157 2044484

[B14] Marsot-Dupuch K Dominguez-Brito A Ghasli K Chouard CH CT and MR findings of Michel anomaly: inner ear aplasia Am J Neuroradiol 1999 20 2 281 284 10094354 PMC7056116

[B15] Daneshi A Farhadi M Asghari A Emamjomeh H Abbasalipour P Hasanzadeh S Three familial cases of Michel's aplasia Otol Neurotol 2002 23 3 346 348 10.1097/00129492-200205000-00020 11981393

[B16] Ramsebner R Ludwig M Parzefall T Lucas T Baumgartner WD Bodamer O Cengiz FB Schoefer C Tekin M Frei K A FGF3 mutation associated with differential inner ear malformation, microtia, and microdontia Laryngoscope 2010 120 2 359 364 19950373 10.1002/lary.20689

[B17] Alvarez Y Alonso MT Vendrell V Zelarayan LC Chamero P Theil T Bosl MR Kato S Maconochie M Riethmacher D Requirements for FGF3 and FGF10 during inner ear formation Development 2003 130 25 6329 6338 10.1242/dev.00881 14623822

[B18] Hatch EP Noyes CA Wang X Wright TJ Mansour SL Fgf3 is required for dorsal patterning and morphogenesis of the inner ear epithelium Development 2007 134 20 3615 3625 10.1242/dev.006627 17855431 PMC2366212

[B19] Mansour SL Goddard JM Capecchi MR Mice homozygous for a targeted disruption of the proto-oncogene int-2 have developmental defects in the tail and inner ear Development 1993 117 1 13 28 8223243 10.1242/dev.117.1.13

[B20] Wilkinson DG Peters G Dickson C McMahon AP Expression of the FGF-related proto-oncogene int-2 during gastrulation and neurulation in the mouse EMBO J 1988 7 3 691 695 3293998 10.1002/j.1460-2075.1988.tb02864.x PMC454374

[B21] Khan SY Riazuddin S Tariq M Anwar S Shabbir MI Riazuddin SA Khan SN Husnain T Ahmed ZM Friedman TB Autosomal recessive nonsyndromic deafness locus DFNB63 at chromosome 11q13.2-q13.3 Hum Genet 2007 120 6 789 793 10.1007/s00439-006-0275-1 17066295

[B22] Ahmed ZM Riazuddin S Bernstein SL Ahmed Z Khan S Griffith AJ Morell RJ Friedman TB Riazuddin S Wilcox ER Mutations of the protocadherin gene PCDH15 cause Usher syndrome type 1F Am J Hum Genet 2001 69 1 25 34 10.1086/321277 11398101 PMC1226045

[B23] Ahmed ZM Masmoudi S Kalay E Belyantseva IA Mosrati MA Collin RW Riazuddin S Hmani-Aifa M Venselaar H Kawar MN Mutations of LRTOMT, a fusion gene with alternative reading frames, cause nonsyndromic deafness in humans Nat Genet 2008 40 11 1335 1340 10.1038/ng.245 18953341 PMC3404732

[B24] Du X Schwander M Moresco EM Viviani P Haller C Hildebrand MS Pak K Tarantino L Roberts A Richardson H A catechol-O-methyltransferase that is essential for auditory function in mice and humans P Natl Acad Sci USA 2008 105 38 14609 14614 10.1073/pnas.0807219105 PMC2567147 18794526

[B25] Arnold K Bordoli L Kopp J Schwede T The SWISS-MODEL workspace: a web-based environment for protein structure homology modelling Bioinformatics 2006 22 2 195 201 10.1093/bioinformatics/bti770 16301204

[B26] Yeh BK Igarashi M Eliseenkova AV Plotnikov AN Sher I Ron D Aaronson SA Mohammadi M Structural basis by which alternative splicing confers specificity in fibroblast growth factor receptors P Natl Acad Sci USA 2003 100 5 2266 2271 10.1073/pnas.0436500100 PMC151329 12591959

[B27] Weerda H Classification of congenital deformities of the auricle Facial Plast Surg 1988 5 5 385 388 10.1055/s-2008-1064778 3270622

[B28] Lee J Blaber M Structural basis of conserved cysteine in the fibroblast growth factor family: evidence for a vestigial half-cystine J Mol Biol 2009 393 1 128 139 10.1016/j.jmb.2009.08.007 19683004

[B29] Ortega S Schaeffer MT Soderman D DiSalvo J Linemeyer DL Gimenez-Gallego G Thomas KA Conversion of cysteine to serine residues alters the activity, stability, and heparin dependence of acidic fibroblast growth factor J Biol Chem 1991 266 9 5842 5846 1706340

[B30] Harmer NJ Pellegrini L Chirgadze D Fernandez-Recio J Blundell TL The crystal structure of fibroblast growth factor (FGF) 19 reveals novel features of the FGF family and offers a structural basis for its unusual receptor affinity Biochemistry 2004 43 3 629 640 10.1021/bi035320k 14730967

[B31] Luo Y Lu W Mohamedali KA Jang JH Jones RB Gabriel JL Kan M McKeehan WL The glycine box: a determinant of specificity for fibroblast growth factor Biochemistry 1998 37 47 16506 16515 10.1021/bi9816599 9843417

[B32] Pellegrini L Role of heparan sulfate in fibroblast growth factor signalling: a structural view Curr Opin Structural Biology 2001 11 5 629 634 10.1016/S0959-440X(00)00258-X 11785766

[B33] Rohmann E Brunner HG Kayserili H Uyguner O Nurnberg G Lew ED Dobbie A Eswarakumar VP Uzumcu A Ulubil-Emeroglu M Mutations in different components of FGF signaling in LADD syndrome Nat Genet 2006 38 4 414 417 10.1038/ng1757 16501574

[B34] Ornitz DM Xu J Colvin JS McEwen DG MacArthur CA Coulier F Gao G Goldfarb M Receptor specificity of the fibroblast growth factor family J Biol Chem 1996 271 25 15292 15297 10.1074/jbc.271.25.15292 8663044

[B35] Mohammadi M Olsen SK Ibrahimi OA Structural basis for fibroblast growth factor receptor activation Cytokine Growth F R 2005 16 2 107 137 10.1016/j.cytogfr.2005.01.008 15863029

[B36] Inan UU Yilmaz MD Demir Y Degirmenci B Ermis SS Ozturk F Characteristics of lacrimo-auriculo-dento-digital (LADD) syndrome: case report of a family and literature review Int J Pediatr Otorhinolaryngol 2006 70 7 1307 1314 10.1016/j.ijporl.2005.12.015 16460812

[B37] Gregory-Evans CY Moosajee M Hodges MD Mackay DS Game L Vargesson N Bloch-Zupan A Ruschendorf F Santos-Pinto L Wackens G SNP genome scanning localizes oto-dental syndrome to chromosome 11q13 and microdeletions at this locus implicate FGF3 in dental and inner-ear disease and FADD in ocular coloboma Hum Mol Genet 2007 16 20 2482 2493 10.1093/hmg/ddm204 17656375

[B38] Pirvola U Spencer-Dene B Xing-Qun L Kettunen P Thesleff I Fritzsch B Dickson C Ylikoski J FGF/FGFR-2(IIIb) signaling is essential for inner ear morphogenesis J Neurosci 2000 20 16 6125 6134 10934262 10.1523/JNEUROSCI.20-16-06125.2000 PMC6772592

